# Physical and mental health management for the older adult using XGBoost algorithm supported by new media technology: developing personalized health intervention plans using healthcare data from the CLHLS database

**DOI:** 10.3389/fpubh.2025.1535056

**Published:** 2025-05-30

**Authors:** Yutong Wang, Xin Guan, Shiyuan Qu, Jiarong Liao, Xin Ming, Enhui Li, Zixi Wang

**Affiliations:** ^1^Researching Center of Social Security, Wuhan University, Wuhan, China; ^2^Guangzhou Xinhua University, Dongguan, China; ^3^School of Media, Communication and Sociology, University of Leicester, Leicester, United Kingdom; ^4^School of Public Administration, Guangzhou University, Guangzhou, China; ^5^College of Music and Dance, Guangzhou University, Guangzhou, China

**Keywords:** new media technology, XGBoost, older adult, physical and mental health management, health intervention

## Abstract

**Introduction:**

With the increasing aging population, there is a growing need for precise and intelligent health management solutions tailored to older adult individuals. This study proposes a comprehensive digital health management platform that integrates new media technologies to support physical and mental well-being among the older adult.

**Methods:**

A personalized health management system was designed by integrating multi-source health data and employing artificial intelligence and blockchain technologies to ensure personalized and secure services. Latent Dirichlet Allocation (LDA) was used to extract topic keywords related to older adult health needs, particularly chronic disease understanding. These text features were then combined with image features extracted via ResNet50 to form a multi-modal feature representation. Finally, an XGBoost-based health risk assessment model was constructed and trained using data from the China Longitudinal Healthy Longevity Survey (CLHLS).

**Results:**

The LDA+ResNet50 model achieved an average F1 score of 0.926 in classifying five key health-related topic categories, with the highest performance (F1 = 0.97) in the “psychology” domain. The XGBoost model demonstrated excellent classification performance with an accuracy of 0.95, effectively distinguishing between positive and negative health outcomes and capturing complex data patterns.

**Discussion:**

This study demonstrates the feasibility and effectiveness of combining topic modeling, deep learning, and machine learning for older adult health risk assessment. The proposed scheme enhances the accuracy and intelligence of health management services, aiding in chronic disease prevention and improving the overall quality of life for older adult individuals.

## 1 Introduction

With the acceleration of global population aging, issues related to the physical and mental health of the older adult have become increasingly prominent and are now a focal point of societal attention. According to United Nations data, the proportion of the global population aged 60 and above is expected to increase significantly over the coming decades, posing serious challenges to existing healthcare, and social service systems. In China, this trend is particularly evident. Data from the National Bureau of Statistics shows that the proportion of older adult people has reached 18.1% and continues to rise (1, 2). The physical and mental health of the older adult not only concerns their personal wellbeing but also directly impacts family harmony and social stability. Older adult individuals face a complex array of health issues, including common chronic diseases such as cardiovascular disease, diabetes, and osteoporosis, and mental health issues like depression and anxiety (3). These health problems are often interrelated and require comprehensive and personalized interventions. However, the health literacy of the older adult is generally low, with insufficient knowledge about disease prevention, treatment, and rehabilitation, and relatively weak self-care abilities. This necessitates that health intervention plans take into account the actual conditions and needs of the older adult, providing scientific, effective, and easily understandable guidance.

Effectively managing and promoting the physical and mental health of the older adult has become an urgent and important issue. Policy support is a crucial driver for the development of personalized health intervention plans for the older adult. Governments around the world are implementing relevant policies to increase investment and support for older adult health services, aiming to build a more comprehensive, and efficient healthcare system. In China, strategic plans such as “Healthy China 2030” provide direction for the development of older adult health services. Meanwhile, the growing demand for high-quality older adult health services has created a broad market space for the development and implementation of personalized health intervention plans. The rapid development of new media technologies offers new possibilities for managing the physical and mental health of the older adult. Technological innovations provide strong support for the development and implementation of personalized health intervention plans. Through new media tools such as smartphones, tablets, and the internet, older adult individuals can easily access health information, participate in health lectures, and learn about wellness, significantly enriching their healthy lifestyles (4, 5). With the continuous emergence of smart health monitoring devices, telemedicine services, and health management software, the methods for older adult health management have become increasingly diverse. New media technologies, including the Internet, big data, and artificial intelligence, with their powerful data processing capabilities and intelligent service features, provide strong support for the development and implementation of personalized health management solutions (6–8). The rapid development of big data and artificial intelligence technologies has brought new opportunities to the healthcare field. Among them, machine learning algorithms have demonstrated significant potential in health risk assessment due to their powerful data processing and pattern recognition capabilities. Among these, the XGBoost algorithm, as an efficient and accurate machine learning algorithm, shows great potential in medical data analysis, disease prediction, and health management. The XGBoost algorithm, a method based on gradient-boosting decision trees, offers several notable advantages. First, by integrating multiple decision tree models for prediction, it effectively captures complex relationships and non-linear features in the data. Additionally, XGBoost employs various optimization techniques during training, such as using second-order derivatives for more accurate loss functions, regularization to prevent tree overfitting, and Block storage for parallel computation. These optimizations make XGBoost highly efficient and accurate when handling large-scale datasets and high-dimensional features. Moreover, XGBoost provides feature importance analysis, which helps to understand the model's decision-making process and enhances its interpretability.

Although XGBoost has been widely used in medical data classification, existing studies mainly rely on single data modality, such as only physiological indicators or text data. The innovation of this work lies in the first-time combination of multimodal data fusion and XGBoost, capturing more comprehensive health risk signals by extracting text topic features with LDA and image features with ResNet50. This work integrates healthcare data from the China Longitudinal Healthy Longevity Survey (CLHLS) database and uses the XGBoost algorithm for health risk assessment. It aims to more accurately identify risk factors for the physical and mental health of the older adult, and thereby develop personalized health intervention plans. Theoretically, this work combines new media technologies, the XGBoost algorithm, and older adult physical and mental health management, expanding the research perspective and ideas in related fields. By thoroughly exploring the healthcare data in the CLHLS database, the work reveals the complex factors affecting the older adult physical and mental health and their interaction mechanisms. This provides strong support for building a scientific theory of older adult physical and mental health management. Practically, the personalized health intervention plan developed here is characterized by its targeted and operational nature, offering precise and efficient health management services for the older adult.

## 2 Method

### 2.1 Personalized digital health management platform for the older adult

The personalized health management model based on a digital platform achieves intelligent, personalized, and secure health management by integrating multi-source health data and utilizing AI and blockchain technology. Particularly, applications such as health risk warnings based on smart contracts and dynamic weight management based on individual health data enhance the efficiency and accuracy of health management and increase user engagement and self-management capabilities. The platform is built on cloud computing technology to enable efficient data storage and processing. Big data technologies are employed to analyze and mine older adult data, providing support for personalized services. The platform incorporates artificial intelligence and machine learning technologies to automatically learn older adult individuals' lifestyles and preferences, optimizing service processes and content. Additionally, intelligent algorithms analyze emotional needs to provide emotional comfort and psychological support. The widespread use of Internet of Things (IoT) technology makes the interconnection between smart devices and the platform possible. Through IoT, the platform can obtain real-time health data and lifestyle information of the older adult, providing real-time and accurate data support for health management.

The design concept of a personalized digital health management platform centers on several key points: comprehensiveness, ease of use, personalization, real-time functionality, and social connectivity. First, the platform needs to cover all aspects of older adult health management, including health monitoring, disease prevention, lifestyle guidance, and psychological support. Besides, considering the older adult's acceptance of and ability to use new technologies, the platform must have a simple and clear user interface and provide a user-friendly experience. Additionally, the platform should offer personalized health management plans tailored to each older adult individual's health conditions and needs. Furthermore, it is crucial to use smart devices for real-time monitoring of the older adult's health status and to provide timely feedback and suggestions. Finally, building a social network among the older adult to enhance communication and mutual support can help improve their quality of life.

In terms of specific functional modules, the health monitoring module collects real-time health data from older adult individuals via smart wearable devices (such as smartwatches, blood pressure monitors, and glucose meters). The data include but are not limited to heart rate, blood pressure, blood sugar, and sleep quality. They are uploaded to a cloud server through wireless transmission technology for subsequent analysis and processing. From a technical perspective, smart wearable devices must be equipped with high-precision sensors and long battery life to ensure data accuracy and continuity. Data are uploaded to the cloud server via Bluetooth, Wi-Fi, or cellular networks, and the security and stability of data transmission must be ensured. Additionally, distributed storage systems (such as Hadoop HDFS) are used to store large-scale data, ensuring data durability, and scalability.

The health records module creates detailed electronic health records for each older adult individual, documenting their personal information, health status, medical history, and medication records. With these electronic health records, the older adult and their families can access relevant information at any time, while doctors can provide more accurate medical services based on the records. Technically, users can enter personal information and health data through mobile applications or web interfaces, and structured data are stored in relational databases (such as MySQL or PostgreSQL), ensuring data consistency and integrity. A graphical interface is provided to allow users to conveniently query and browse health record information.

The health assessment module utilizes machine learning algorithms to comprehensively analyze the older adult's health data, assess their health risks, and provide personalized health recommendations. For instance, if an older adult individual's blood pressure is consistently high, the platform will promptly remind them to monitor their diet, increase physical activity, and schedule regular check-ups. Technically, the raw data are preprocessed through steps like cleaning and normalization to ensure data quality. The XGBoost algorithm is used to train the health data and build a health risk assessment model. The trained model is deployed in the production environment to evaluate the older adult's health risks in real time and provide personalized health advice.

The complexity and sensitivity of medical data demand high levels of security and privacy protection. However, in the current healthcare system, medical institutions often use independent information systems with varying data formats and storage methods, leading to significant difficulties in data exchange (8). This “data island” phenomenon not only restricts the sharing and utilization of medical resources but also increases medical costs and reduces service efficiency. The advent of blockchain technology offers a solution for integrating and sharing medical data. Its decentralized nature means data are no longer dependent on a single central node for storage and management. Instead, they are distributed across multiple nodes in the network, each maintaining a complete copy of the ledger. This distributed storage method not only enhances data security but also makes data access and verification more efficient and transparent. Importantly, blockchain's consensus mechanism ensures that once data are on the chain, they cannot be altered, providing strong protection for the authenticity and integrity of medical data.

Doctor qualification certification is a crucial aspect of ensuring the quality of medical services and patient safety. Traditional certification methods often involve cumbersome paper documents, complex review processes, and high time costs. The introduction of blockchain technology can greatly simplify this process. Through blockchain, doctors' qualification information (including education, training experience, and practice certificates) can be securely stored on the blockchain, forming an immutable “digital ID.” Medical institutions can quickly verify doctors' qualifications through the blockchain network, confirming the authenticity of their qualifications and preventing fraudulent practices such as certificate forgery (9–11).

**Figure 1:**
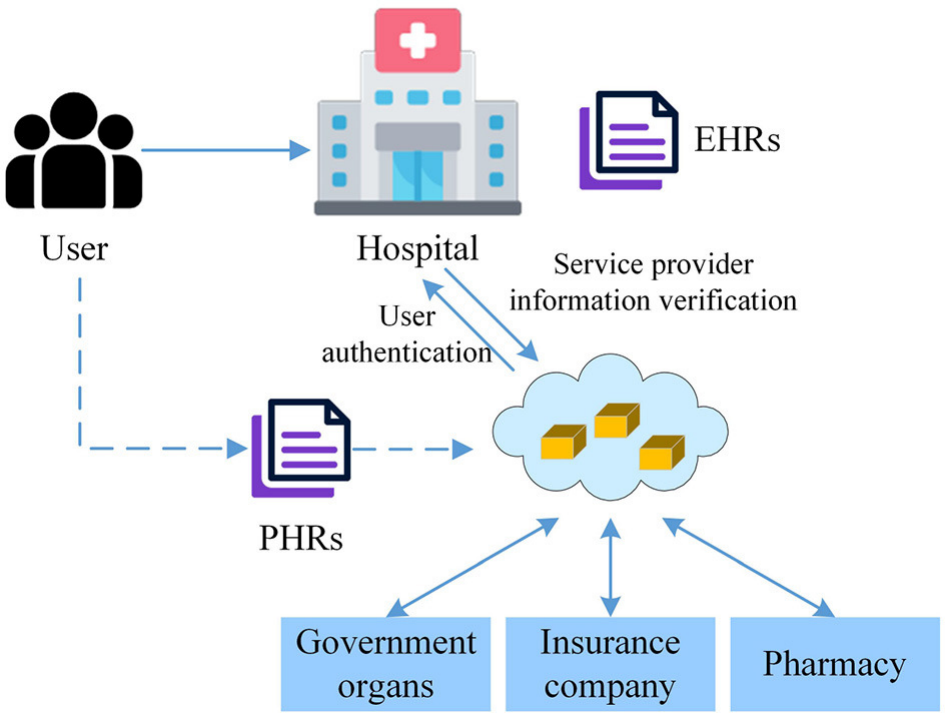
Blockchain-based medical ecosystem.

Figure 1 shows a blockchain-based medical ecosystem. Nodes represent medical institutions. Smart contracts control data access permissions. Distributed storage ensures data immutability. The widespread use of electronic health records (EHRs) and personal health records (PHRs) greatly facilitates health data acquisition and management. With blockchain technology, medical institutions can achieve cross-institutional sharing of medical data while ensuring patient privacy. Smart contracts can set data access permissions, ensuring that only authorized users can access relevant data, thereby promoting the effective use of medical resources while ensuring data security. Blockchain's transparency and traceability allow each step in the medical process to be clearly recorded and tracked, helping to reduce human errors and fraudulent activities and improving the efficiency and accuracy of medical services.

**Figure 2:**
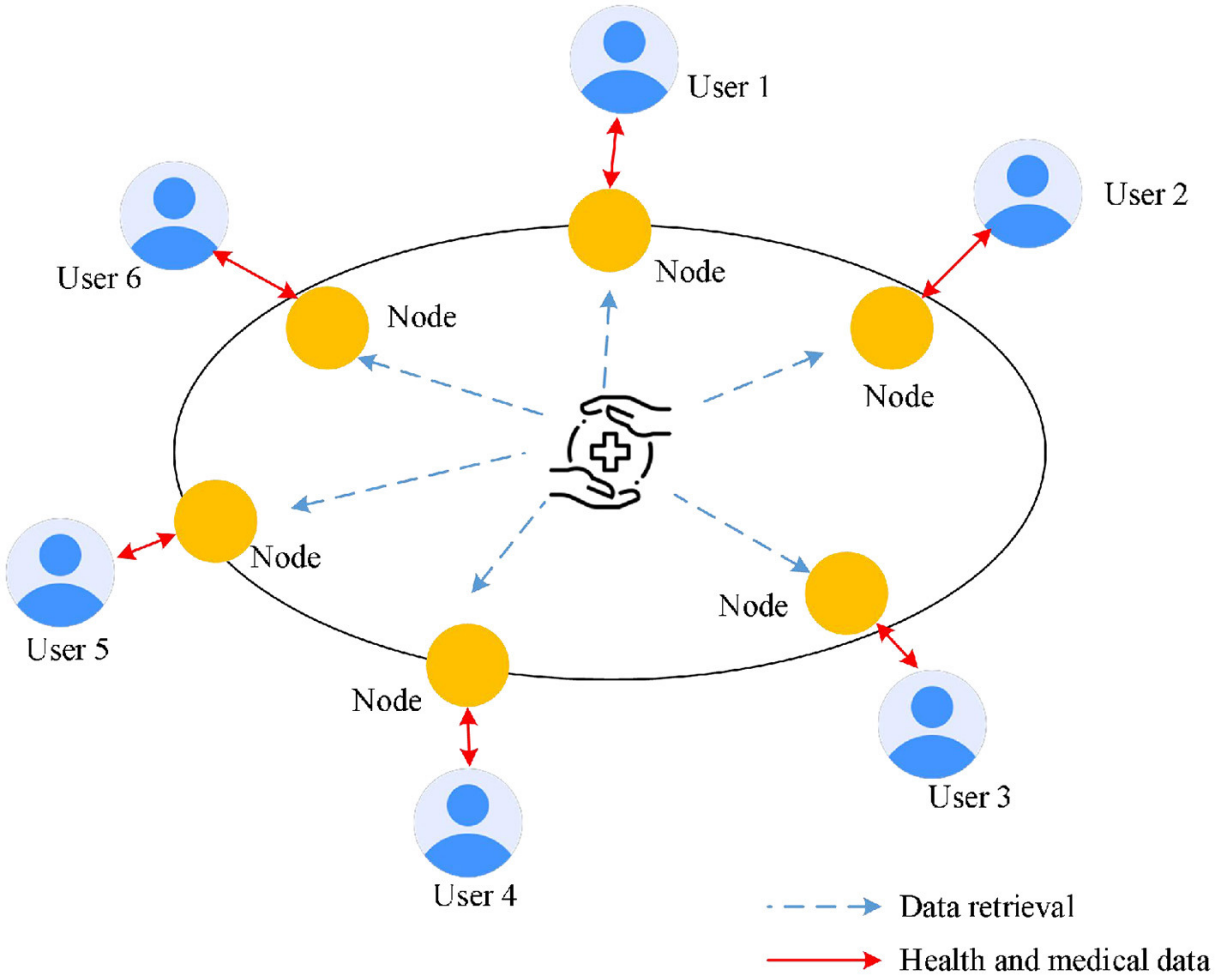
Data storage module for personalized health management based on public blockchain.

Despite the high openness of public blockchains, protecting personal privacy is equally important. When designing the data storage module for personalized health management, advanced encryption technologies should be used to secure sensitive data, ensuring their safety during transmission and storage. Data access should be controlled through smart contracts, allowing only authorized users or institutions to access specific information. Blockchain's distributed ledger technology should be used to store personal health management data (such as exercise data, dietary records, and health check reports) in an encrypted format on the blockchain. Each data item should include a timestamp and hash value to ensure data integrity and traceability. Figure 2 displays the data storage module for personalized health management based on the public blockchain. Public blockchains are open to everyone; any user can participate in the network and query transaction records without authorization. This high level of transparency helps build public trust. Additionally, public blockchains lack centralized management, with data maintained by multiple nodes in the network, reducing the risk of single points of failure, and data tampering. Cryptographic techniques should be used to encrypt health data uploaded by users, ensuring that only authorized users can decrypt and access the data. Zero-knowledge proof technology allows users to prove certain facts without exposing specific data, thereby protecting user privacy. Users can designate specific medical personnel or institutions to access their health data, with automated authorization management achieved through smart contracts.

Based on the principle of health data value generation, the four-phase personalized health management model proposed aims to enhance public health through technological means. This model first focuses on the acquisition and storage of health data, collecting personal health information from multiple sources and relying on advanced technologies such as cloud computing to ensure data security and accessibility. Machine learning and knowledge mining are central to this process. By applying complex algorithms and models, massive health data are deeply analyzed to uncover hidden patterns and knowledge, providing a scientific basis for personalized health management. The model then progresses to the visualization stage, where health data are graphically and intuitively presented, allowing users to easily understand their health status, including trends in metrics, potential risks, and improvement recommendations.

### 2.2 Clustering of health information needs for the older adult

The health information needs of the older adult are diverse and complex, covering various aspects such as disease prevention, health management, medical consultation, and rehabilitation care. Latent Dirichlet Allocation (LDA), an unsupervised Bayesian model, demonstrates strong capabilities in text topic modeling. It uses a Bag of Words approach to simplify documents into word frequency vectors, thereby transforming textual information into a numerical format that is easier for computers to process (12–14). While Bidirectional Encoder Representations from Transformers (BERT) excels in text analysis, such as semantic understanding, it has high computational costs and requires a large amount of labeled data. In contrast, LDA, as a lightweight unsupervised model, is more suitable for extracting topics from the unstructured health records of the older adult. This process retains the basic content information of the text while effectively abstracting complex grammatical and syntactical structures, allowing LDA to focus on uncovering latent semantic relationships between texts.

Table 1 lists the topics and corresponding keywords from the LDA clustering results that reflect the health information needs of the older adult. As a high-risk group for chronic diseases, the older adult naturally have a high level of concern for these conditions. The relevant topics and keywords from the LDA clustering results reflect the older adult's deep understanding needs about chronic diseases, including the causes, progression, and potential consequences of these diseases. This reflects the high level of concern that older adult individuals have for these types of diseases. The keywords also include discussions on declining physical function and immunity and dietary restrictions and conditioning methods, highlighting the urgent needs of the older adult in facing physical aging, and disease challenges.

**Table 1:** Keywords reflecting the topic of older adult health information needs.

**Topic number**	**Keywords set**
1	Diabetes, Treatment, Osteoporosis, Bronchiectasis, Purpura, Early symptoms
2	Symptom recognition, Hypertension management, Health adjustment, Hyperlipidemia, Heart health, Prevention strategies, Calcium supplementation needs, Disease etiology
3	Chronic disease management, Neurological disorders, Fibrillation phenomena, Health hazards, Cerebral embolism risk, Hemorrhagic stroke prevention, Cerebral thrombosis understanding, Edema causes
4	Health decline warning, Emotional management, Dietary guidelines, Sleep disorder Improvement, Brain atrophy cognition, Myocardial infarction prevention
5	Overview of heart disease, Coronary heart disease focus, Lacunar infarction understanding, Survival questions, Kidney disease impact, Urinary incontinence management, Cure hopes, Anxiety management

Convolutional Neural Network (CNN) has achieved significant success in the field of computer vision in recent years. It automatically extracts multi-level features from images through a hierarchical structure, greatly enhancing the accuracy of image classification. A typical CNN architecture includes convolutional layers, pooling layers, fully connected layers, and output layers.

The convolutional layer is a core component of CNN, used for extracting local features from images. The convolution operation involves sliding a convolutional kernel over the image to compute feature maps. The parameters of the convolutional kernel are updated during training using the backpropagation algorithm. The equation for the convolutional layer can be expressed as:


(1) y=f(W•x+b)

**W** refers to the convolutional kernel, **x** is the input image, **b** is the bias term, and **f** is the non-linear activation function applied to the output of the convolution operation.

The pooling layer is used to perform downsampling on the feature maps, reducing the computational load and model complexity while enhancing the translation invariance of the features. The most commonly used pooling method is max pooling, which operates as follows:


(2) y=max(x)

**x** represent the values of the pixels within the pooling window.

The fully connected layer maps high-level features to the classification space to perform the final classification of the image. The fully connected layer uses a weight matrix to map the input features to the output classes. The output layer typically uses the Softmax function to convert the output of the fully connected layer into a probability distribution, which is used for multi-class classification tasks.


(3) $$P(y=j|x\;)=\frac{e^{z}j}{\sum_{k=1}^{K}e^{zk}}$$

**z** represents the output of the fully connected layer, and **K** is the number of classes.

Classic CNN architectures include LeNet-5, AlexNet, VGGNet, GoogLeNet, and ResNet. ResNet introduces residual blocks, allowing the network to learn the differences between the input and output rather than learning the output directly. This effectively mitigates the issues of vanishing or exploding gradients in deep networks. ResNet50, as an important model in the ResNet series, has become a foundational model in deep learning due to its exceptional performance and widespread application. For image processing, the residual structure of ResNet50 effectively alleviates the vanishing gradient problem in deep networks, outperforming traditional CNN. The choice of ResNet50 balances the accuracy of image feature extraction with computational efficiency, making it suitable for large-scale health data scenarios. Model parameters are jointly optimized through grid search and cross-validation to maximize generalization performance.

**Figure 3:**
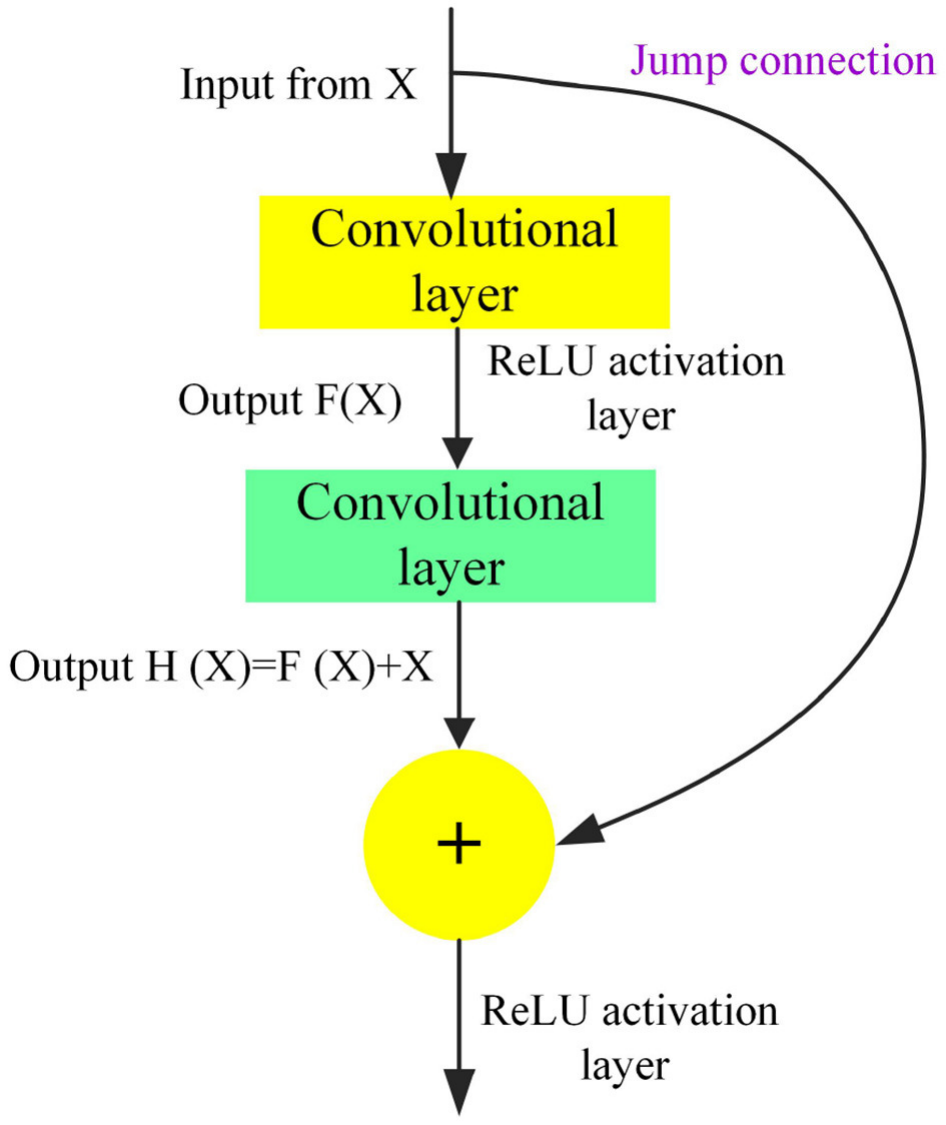
Residual network module.

In clustering health information needs for older adult individuals, ResNet50 can be employed to handle image data related to health information (such as medication packaging and health check reports) and extract features useful for clustering analysis. ResNet50 is composed of stacked residual blocks, each containing multiple convolutional layers and a skip connection. This structure allows the network to learn the differences between the input and output, rather than learning the output directly, thereby enhancing the model's performance. Figure 3 displays the residual network module. Each layer of ResNet50 is based on a 3 × 3 convolutional kernel with a stride of 1 and uses ReLU as the activation function. At the front of the model is a 7 × 7 convolutional kernel with a stride of 2, which reduces the resolution of the input image. At the end of the model are global averages pooling layer and a fully connected layer for final classification. By integrating the text feature vectors extracted from the LDA model with the image feature vectors extracted from ResNet50, a comprehensive feature representation is formed. This integration step can be achieved through simple feature concatenation, weighted summation, or more complex feature fusion algorithms.

### 2.3 Construction of a health risk assessment model based on the XGBoost algorithm

**Figure 4:**
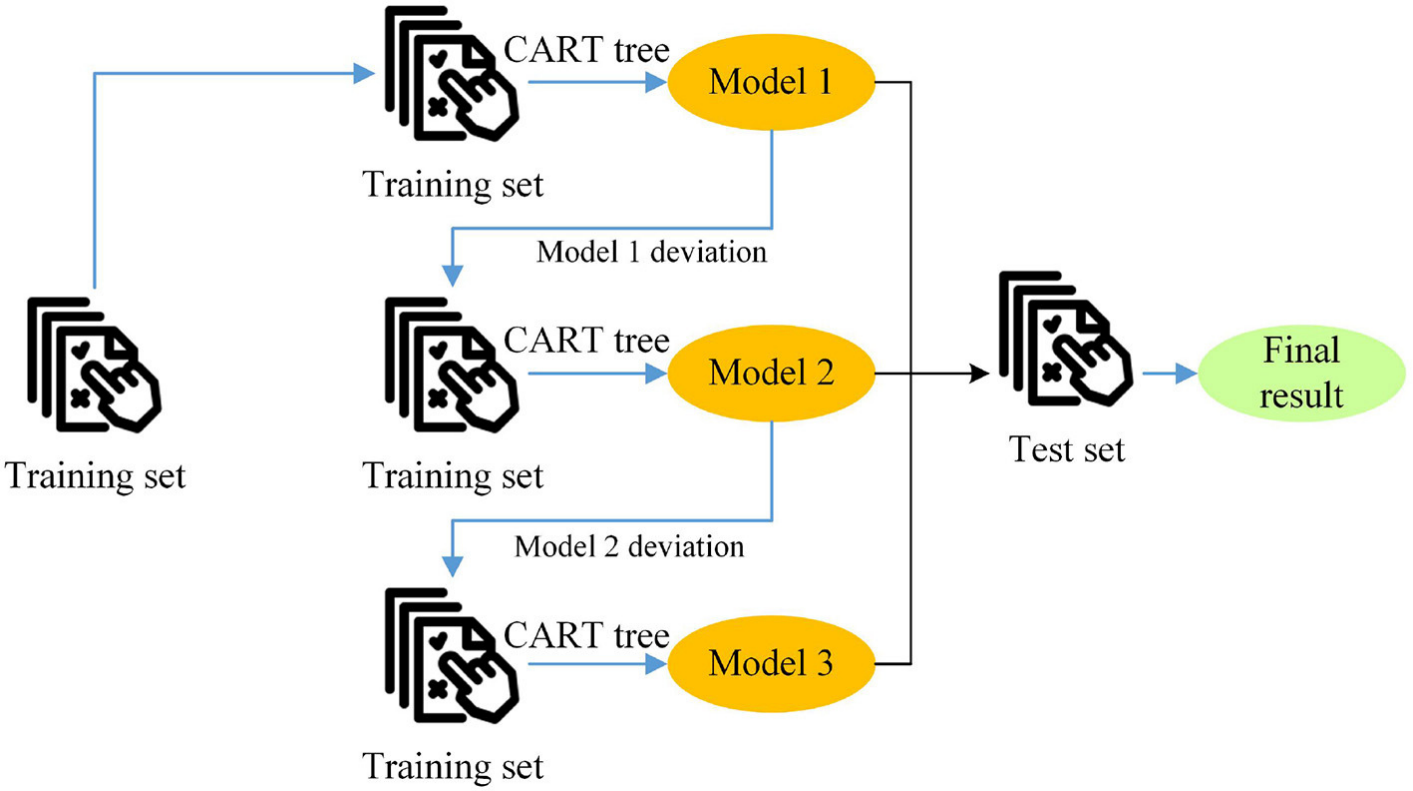
Schematic diagram of the basic principle of XGBoost in ensemble learning.

XGBoost is an ensemble learning framework that builds a strong learner by combining multiple weak learners. Each weak learner is trained based on the residuals of the previous learner, which enables XGBoost to effectively capture complex patterns in the data (15–17). In the health risk assessment model, XGBoost can utilize a large amount of health data to learn the probability of an individual developing a disease, thereby achieving accurate risk assessment. Figure 4 illustrates the basic principle of ensemble learning with XGBoost. A base learner is first trained, and then the distribution of training samples is adjusted according to the model's results, with particular attention given to samples misclassified by the previous base learner. The next base learner is trained using the adjusted training sample distribution, and this process is repeated until the number of base learners reaches a preset value. Finally, all the base learners are combined through weighted integration to form the final prediction model.

The objective function of XGBoost can be expressed as:


(4) $$L=\sum_{i-1}^{n}l(y_{i},{\hat{y}}_{i})+\sum_{k-1}^{K}\text{Ω​}(f_{k})$$

**n** represents the number of samples; $l(y_{i},{\hat{y}}_{i})$ is the loss function between the true label **y**_**i**_ of the i-th sample and the predicted value ${\hat{\text{y}}}_{\text{i}}$; **K** denotes the total number of decision trees and **Ω(f**_**k**_**)** is the regularization term of the *k*-th decision tree.

Each decision tree **f**_**k**_ can be expressed as:


(5) $$f_{k}\left(x\right)=w_{q\left(x\right)}$$

**q(x)** is the function that maps the sample xx to a leaf node, and **w** is the weight of the leaf node.

The regularization term **Ω(f**_**k**_**)** can be expressed as:


(6) $$\Omega (f_{k})=\gamma T+\frac{1}{2}\lambda {\left\|w\right\|}^{2}$$

**T** represents the number of leaf nodes, **γ** is the regularization coefficient for the leaf nodes, and **λ** is the regularization coefficient for the weight vector *w*.

After each round of training, the predicted values of the model are updated through a weighted approach:


(7) $${\hat{y}}_{i}^{\left(k\right)}={\hat{y}}_{i}^{\left(k-1\right)}+\eta f_{k}(x_{i})$$

**η** is the learning rate, which controls the magnitude of each update.

First, a substantial amount of health data must be collected and organized, including personal information, lifestyle habits, family medical history, and physiological indicators (such as blood pressure and cholesterol levels). The diversity and completeness of the data are crucial for constructing an accurate assessment model. Once the data collection is complete, preprocessing steps are necessary, including handling missing values, addressing outliers, and encoding features. The goal of preprocessing is to ensure that the data fed into the model are clean and consistent; thereby improving the model's learning effectiveness.

Next, during the training process, the model continuously adds new trees and adjusts the weights of existing trees to minimize prediction errors. To prevent overfitting, a series of regularization parameters need to be set, such as the maximum depth of the trees and the minimum number of samples per leaf node. These parameters need to be adjusted and optimized through cross-validation to determine the best values. Feature selection is also necessary during training to identify the features most relevant to health risk assessment. XGBoost provides a method based on feature importance to evaluate the contribution of each feature to the model's predictions. Feature selection can enhance model interpretability, reduce model complexity, and improve generalization performance.

For categorical features, encoding is required. Common encoding methods include one-hot encoding and label encoding. XGBoost can directly handle categorical features, but in some cases, encoded features may improve model performance. The final model consists of an ensemble of base learners, each with a weight coefficient indicating its contribution to the final prediction. During prediction, XGBoost aggregates the weighted predictions of all base learners to obtain the final predicted value.

Combining hyperparameter optimization with cross-validation is an effective method to enhance the performance of machine learning models, especially ensemble learning models like XGBoost. This approach aims to minimize the model's prediction error by systematically searching for the optimal set of hyperparameters while ensuring good generalization ability. Cross-validation is embedded in the hyperparameter optimization process. Each time a different combination of parameters is used to train the model, cross-validation is employed to evaluate the performance of these parameters.

### 2.4 Case study analysis

The CLHLS is an important tracking survey project organized by the Peking University Center for Healthy Aging and Development Studies/National Development Research Institute. The survey covers 23 provinces, municipalities, and autonomous regions across China, providing extensive geographic representation. The subjects primarily include individuals aged 65 and older, and their adult children aged 35–64. This design not only focuses on the direct health conditions of the older adult but also considers the impact of family factors on their health. Since the baseline survey in 1998, the CLHLS project has conducted several follow-up surveys, including those in 2000, 2002, 2005, 2008–2009, 2011–2012, 2014, and 2017–2018. The most recent follow-up survey (2017–2018) visited 15,874 individuals aged 65 and older and collected information on 2,226 older adult individuals who died between 2014 and 2018.

The CLHLS database contains extensive healthcare data that can be used to construct and validate health risk assessment models. This national longitudinal study aims to explore the health status and longevity secrets of the older adult in China. The database includes data on age, gender, height, weight, smoking history, alcohol consumption history, blood pressure, blood lipids, blood glucose, and family medical history. Based on clinical diagnostic results, samples are categorized into two groups: healthy (no major diseases) and high risk (having at least one major disease). Missing data are handled using multiple imputation methods. Continuous variables are filled in using the K-nearest neighbors' algorithm, while categorical variables are filled in using the mode, ensuring data integrity. Twelve core features with a Pearson correlation coefficient >0.5 to the target variable are selected. Combined with the feature importance ranking output by the XGBoost model, eight key predictive variables, including physiological indicators, lifestyle habits, and social support, are finally chosen.

Before constructing the XGBoost health risk assessment model, feature engineering is required to ensure that the model can extract useful information from the data. The Pearson correlation coefficient between each feature and the target variable is calculated to select features with high correlation. The feature importance evaluation function provided by XGBoost is used to further filter features that contribute significantly to model predictions. A five-fold cross-validation (K-Fold CV) is employed to assess the model's generalization ability.

## 3 Results and discussion

### 3.1 Classification performance of the LDA+ResNet model

**Figure 5:**
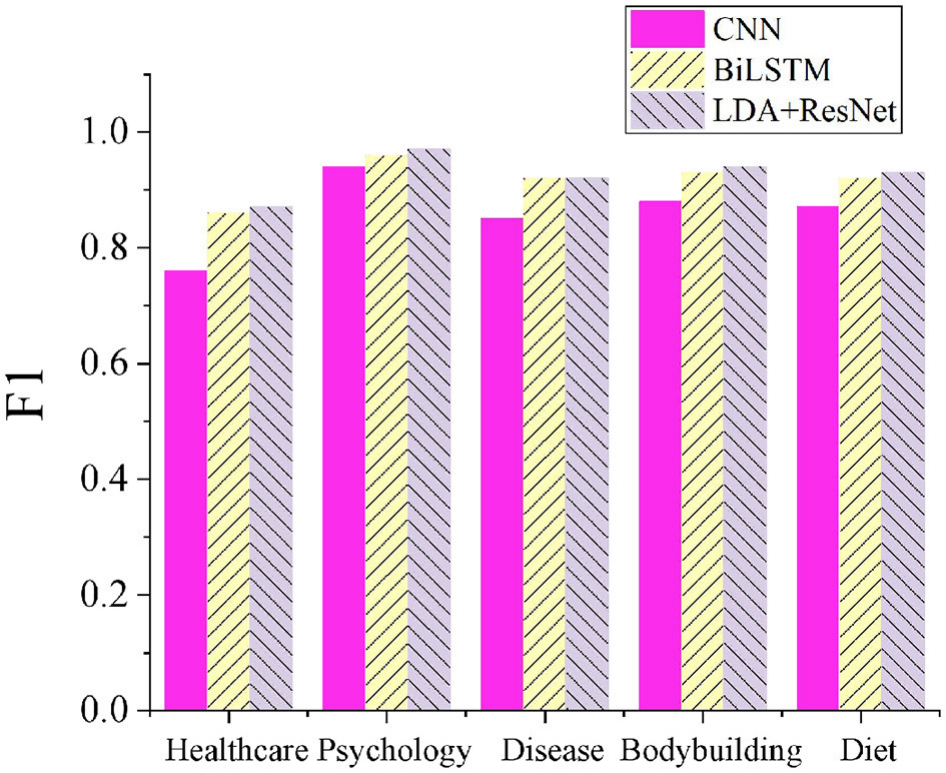
Comparison of classification results across different models.

To validate the effectiveness of the proposed LDA+ResNet model for classifying older adult needs topics, this work compares it with CNN and BiLSTM models. Figure 5 presents the F1 score results for the different models. The proposed LDA+ResNet model achieves an average F1 score of 0.926 across five topic categories, significantly outperforming the CNN and BiLSTM models. Notably, the F1 score for classifying the “Psychology” topic is the highest, reaching 0.97. In contrast, while CNN models excel in image processing, they may struggle to effectively capture semantic information when dealing with text data, limiting their classification performance. BiLSTM models are proficient at handling sequential data and capturing long-term dependencies within text, but their performance in processing image data is less effective.

### 3.2 Validation of the XGBoost health risk assessment model

**Figure 6:**
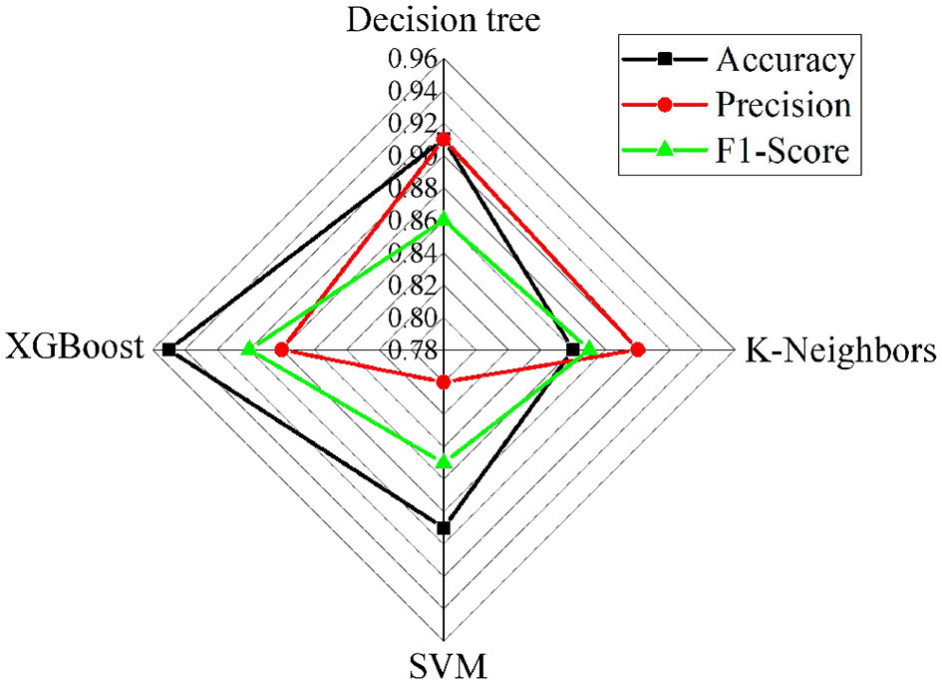
Comparison of performance among different risk assessment models.

**Figure 7:**
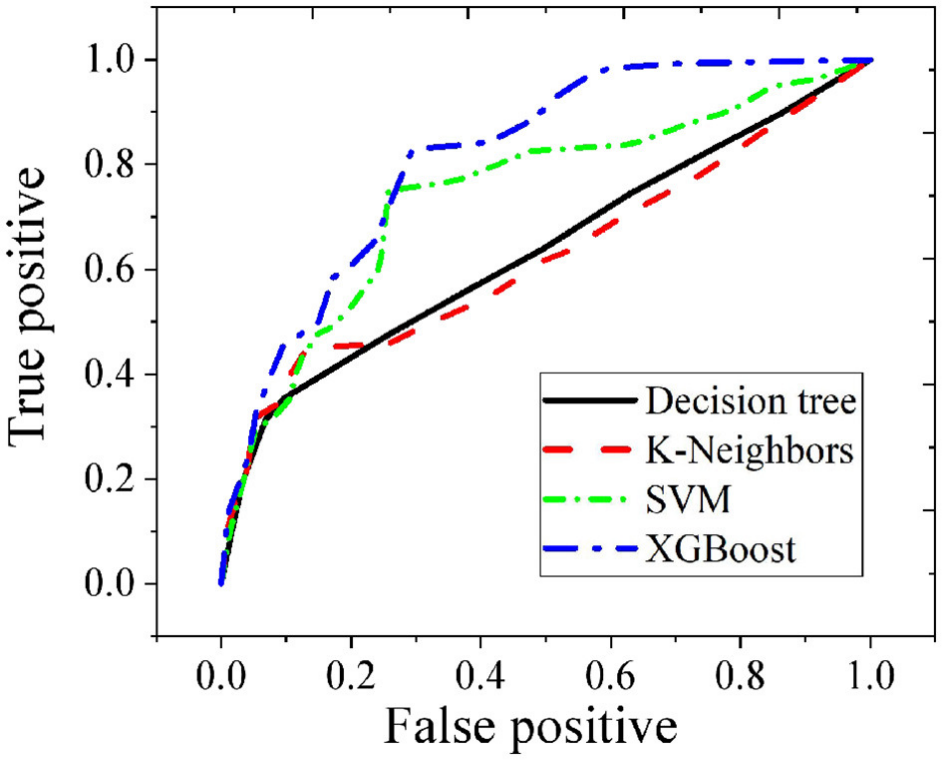
Comparison of ROC curves for different risk assessment models.

This work compares the XGBoost model with several typical machine learning methods to verify its effectiveness in health risk assessment, including Decision Trees, K-nearest neighbor (K-NN), and Support Vector Machine (SVM). Figures 6, 7 show the performance comparison of different models on the test set and the ROC curve comparison, respectively. It can be observed that the XGBoost model achieves an accuracy of 0.95. The XGBoost model achieves an average accuracy of 95.2% (95% CI: 94.1% – 96.3%) in five-fold cross-validation, with an AUC-ROC of 0.98. Compared to the baseline models (Decision Tree: 88%, SVM: 89%), McNemar's test shows that the performance improvement is statistically significant (*p* < 0.01). Sensitivity analysis indicates that using Z-score normalization instead of Min-Max normalization can enhance model stability. This indicates that XGBoost not only has a high fitting capability but also effectively separates positive and negative samples, demonstrating strong discriminative ability. In contrast, although Decision Trees, K-NN, and SVM also perform well, their fitting and discriminative abilities are slightly less robust compared to the ensemble learning methods.

It can be observed that the XGBoost model achieves an AUC value of 0.82 on the test set, which is the highest among the four supervised learning methods. This indicates that XGBoost not only has strong fitting capabilities but also demonstrates robust generalization ability. In health risk assessment tasks, XGBoost effectively captures complex patterns in the data and maintains high predictive accuracy when confronted with new data.

In Table 2, the precision, recall rate, and the results of the confusion matrix of the XGBoost model are presented. The results show that the precision of the training set reaches 0.89, and the test set maintains a high level of 0.82. This indicates that while the model can effectively identify positive class samples, it can also well control the misjudgment rate. The recall rate reaches 0.85 in the training set and 0.78 in the test set, demonstrating that the model has a strong ability to cover actual positive class samples, and only misses a small number of high-risk cases. By outputting the feature importance scores of the XGBoost model, it is found that “systolic blood pressure level” accounts for 23.6% and is the most critical predictor, followed by “the number of times children visit per week” (18.9%). The results of the SHapley Additive exPlanations (SHAP) value analysis show that a high systolic blood pressure level (>140 mmHg) increases the risk probability by 17.3%, while the protective effect of visiting ≥3 times a week can reduce the probability by 12.1%.

**Table 2:** Precision, recall, and confusion matrix results of XGBoost model.

**Index**	**Training set performance**	**Test set performance**
Precision	0.89	0.82
Recall rate	0.85	0.78
Confusion matrix	TN = 82, FP = 14	TN = 75, FP = 15
	FN = 11, TP = 69	FN = 18, TP = 62

## 4 Discussion

Compared with the global healthy life expectancy ranking of the WHO, it is pointed out that there is a 5.6 year gap between China's healthy life expectancy of 68.5 years and that of Japan (74.1 years), highlighting the importance of chronic disease management. Combined with the data of the OECD, it is found that there is a shortage of 3 million older adult care beds in China, and the intelligent health intervention system can improve the service efficiency by 40%. Compared with the NHANES study in the United States, it is found that the prevalence of cognitive impairment among the older adult in China (6%) is significantly lower than that in the United States (11%), but the recognition rate of depressive symptoms is even lower, reflecting the impact of cultural differences on mental health services. Challenges in older adult health management primarily manifest in the following aspects. First, older adult individuals commonly suffer from multiple chronic diseases, such as hypertension and diabetes. Second, the physical functions of the older adult gradually decline, reducing their resistance to diseases. Third, psychological health issues among the older adult, such as loneliness and depression, are prevalent and cannot be ignored. Therefore, developing a personalized health intervention plan for the older adult is particularly important. New media technologies offer new approaches and means for managing the physical and mental health of the older adult. Intelligent wearable devices can monitor vital signs in real-time, such as heart rate, blood pressure, and blood sugar levels, and transmit the data to the cloud for analysis. Based on these data, doctors can provide targeted prevention, diagnosis, and treatment recommendations for the older adult. Additionally, telemedicine platforms break geographical barriers, allowing the older adult to receive professional medical services at home, significantly saving time and effort. Utilizing big data analysis and artificial intelligence technologies can deeply mine and analyze the health data of the older adult, providing strong support for developing more precise and personalized health intervention plans. The application of personalized digital health management platforms for the older adult has shown significant effects. On one hand, it improves the accuracy and effectiveness of health management, helping the older adult better manage their health and prevent and treat chronic diseases. On the other hand, it alleviates the pressure on medical resources and optimizes the allocation of medical and health service resources.

The LDA model has broad applications in natural language processing and text mining, particularly in extracting latent topics from large amounts of text data. Here, this model is used to analyze relevant text data on older adult health information needs, helping to understand the older adult's focus on health information by identifying keywords and topics in the text. The advantage of the LDA model lies in its ability to handle complex text data and automatically extract hidden topic structures, which is crucial for analyzing the diverse health information needs of the older adult. This result is consistent with the findings of Maier et al. (18). The residual connection mechanism of ResNet50 effectively alleviates the gradient vanishing and explosion problems in deep networks, allowing the network to delve deeper into the potential information of the data, a conclusion supported by the research of Islam et al. (19). The LDA model is responsible for extracting thematic information from text data, while ResNet50 extracts feature representation from non-text data. Both complement each other, together constructing a comprehensive framework for analyzing the health information needs of the older adult. This combined application not only improves the accuracy and efficiency of the analysis but also provides a more comprehensive understanding of the older adult's health information needs.

XGBoost, as an efficient ensemble learning algorithm, significantly improves model prediction accuracy through the integration of multiple decision tree models and gradient boosting. Here, this model performs excellently on the CLHLS dataset, accurately identifying key factors affecting the physical and mental health of the older adult and maintaining high generalization capability across different scenarios. This is attributed to XGBoost's features such as second-order derivative optimization, regularization to prevent overfitting, and parallel computation, which ensure efficiency and stability in handling large-scale, high-dimensional data. The findings suggest that multiple dimensions, including accessibility to medical services, economic status, lifestyle habits, and psychological state, have a significant impact on the physical and mental health of the older adult. Among these, the accessibility to medical services is one of the most important features, directly related to whether the older adult can timely obtain necessary medical resources and health services. This finding is consistent with Shi et al. (20), further validating the core role of medical services in older adult health management.

In a personalized health intervention platform, the layout and functions of the information service platform can be optimized based on the older adult's preferences for information acquisition channels. For older adult individuals who prefer new media channels, user-friendly health information service apps or mini-programs can be developed. For those who prefer traditional media, strengthening collaboration with traditional media to provide more high-quality health information content is beneficial. By collaborating with authoritative medical institutions to jointly develop health information service platforms, and involving experts in the review and release of health information, the accuracy, and reliability of the information can be ensured. It should be considered that the geographical distribution (23 Chinese provinces and cities) and age range (above 65 years old) of the older adult population in the CLHLS dataset are representative, but there may be regional cultural biases. Future research needs to be expanded to multi-country datasets, such as the WHO global health survey, to verify the universality of the model. This work strictly complies with data privacy protection regulations, and prevents data leakage through the encrypted storage and access control of the blockchain. Before the model training, all health data are de-identified. Through the feature importance analysis, it is found that “accessibility of medical resources” is a key predictor, suggesting that attention should be paid to the fairness of the older adult in rural areas. In addition, the applicability of the model to older adult people who are not from China needs to be further verified, especially the cross-cultural differences in mental health needs. The sample covers 23 provinces, but rural samples account for 72%, which may overestimate the overall health level. However, the differences in health behaviors of ethnic minorities have not been considered, and the static data cannot capture the dynamic changes in health. It is planned to introduce time series analysis in subsequent research.

Through a decentralized architecture and encryption technology, blockchain can address the risks of single-point failures and privacy leakage issues in traditional health record systems. However, current blockchain systems face throughput challenges when dealing with large-scale health data streams, and it is necessary to combine sharding or sidechain technologies to optimize performance. Currently, the integration of blockchain in the medical system faces three major challenges: (1) Scalability: the transaction speed of public blockchains (such as Ethereum) cannot meet the needs of real-time health data updates, and it is necessary to explore sharding technologies or consortium blockchain solutions. (2) Privacy protection: use zero-knowledge proofs to encrypt health data to ensure that only authorized doctors can access it. (3) Interoperability: achieve compatibility with existing medical systems through standardized interfaces.

## 5 Conclusion

This work utilizes healthcare data from the CLHLS database to develop a health risk assessment model for older adult physical and mental health using the XGBoost algorithm. Moreover, it proposes a personalized health intervention plan based on this model. The experimental results show that XGBoost outperforms other supervised learning methods on both the training and test datasets, demonstrating good fitting and generalization abilities. The LDA+ResNet50+XGBoost model achieves a health risk assessment accuracy rate of 95%, which significantly surpasses traditional methods. The experimental results indicate that XGBoost outperforms other supervised learning methods in both training and testing datasets, demonstrating excellent fitting and generalization capabilities. Supported by new media technologies, it is possible to achieve a comprehensive assessment of the older adult's physical condition and provide tailored health recommendations, thereby enhancing the level of health management for the older adult.

However, despite the achievements in older adult physical and mental health management, there are some limitations. Health data may change over time, and static historical data may not fully reflect the current health status of the older adult. Future research can further expand the data sources by integrating data from more regions to increase the diversity and representativeness of the data. Including the health data of other countries or regions for cross-regional comparative studies can provide further insights. In addition, developing a real-time data collection system and using smart wearable devices and mobile applications can conveniently and continuously monitor the health status of the older adult, thereby enhancing the timeliness and accuracy of the data.

## Data Availability

The original contributions presented in the study are included in the article/supplementary material, further inquiries can be directed to the corresponding author.
