# Correction: Physical and mental health management for the older adult using XGBoost algorithm supported by new media technology: developing personalized health intervention plans using healthcare data from the CLHLS database

**DOI:** 10.3389/fpubh.2025.1642584

**Published:** 2025-06-27

**Authors:** Yutong Wang, Xin Guan, Shiyuan Qu, Jiarong Liao, Xin Ming, Enhui Li, Zixi Wang

**Affiliations:** ^1^Researching Center of Social Security, Wuhan University, Wuhan, China; ^2^Guangzhou Xinhua University, Dongguan, China; ^3^School of Media, Communication and Sociology, University of Leicester, Leicester, United Kingdom; ^4^School of Public Administration, Guangzhou University, Guangzhou, China; ^5^College of Music and Dance, Guangzhou University, Guangzhou, China

**Keywords:** new media technology, XGBoost, older adult, physical and mental health management, health intervention

In the published article, there was a mistake in the Funding statement. The study did not receive any financial support, contrary to the funding statement currently included. The correct funding statement reads:

“The author(s) declare that no financial support was received for the research, authorship, and/or publication of this article.”

The original article has been updated.

